# Supplementary material for: Modeling glioblastoma heterogeneity as a dynamic network of cell states
Source: Mol Syst Biol. 2021 Sep 16;17(9):e10105. doi: 10.15252/msb.202010105 (PMC8444284; doi:10.15252/msb.202010105)
Supplement: Supplementary file 5 — Source Data for Figure 3 [file MSB-17-e10105-s001.zip › Figure3A_sourcedata/GSEA_3065/nosigdb_state6.GseaPreranked.1622718443993/RICHARDS_NOWAKOWSKI_SCIENCE_2017_IPC_NEN3_UPREG.html]

Details for gene set RICHARDS\_NOWAKOWSKI\_SCIENCE\_2017\_IPC\_NEN3\_UPREG[GSEA]

|  || Dataset | state6 |
| Phenotype | NoPhenotypeAvailable |
| Upregulated in class | na\_pos |
| GeneSet | RICHARDS\_NOWAKOWSKI\_SCIENCE\_2017\_IPC\_NEN3\_UPREG |
| Enrichment Score (ES) | 0.4663863 |
| Normalized Enrichment Score (NES) | 2.2178707 |
| Nominal p-value | 0.0 |
| FDR q-value | 2.7238094E-4 |
| FWER p-Value | 0.002 |
Table: GSEA Results Summary

  

Fig 1: Enrichment plot: RICHARDS\_NOWAKOWSKI\_SCIENCE\_2017\_IPC\_NEN3\_UPREG      
 Profile of the Running ES Score & Positions of GeneSet Members on the Rank Ordered List

  

| PROBE | GENE SYMBOL | GENE\_TITLE | RANK IN GENE LIST | RANK METRIC SCORE | RUNNING ES | CORE ENRICHMENT || 1 | RPLP1 |  |  | 6 | 0.329 | 0.0144 | Yes |
| 2 | RPLP0 |  |  | 10 | 0.312 | 0.0284 | Yes |
| 3 | RPS27 |  |  | 11 | 0.307 | 0.0425 | Yes |
| 4 | RPS19 |  |  | 14 | 0.295 | 0.0558 | Yes |
| 5 | RPL13 |  |  | 21 | 0.286 | 0.0682 | Yes |
| 6 | RPL3 |  |  | 22 | 0.285 | 0.0813 | Yes |
| 7 | RPLP2 |  |  | 31 | 0.271 | 0.0928 | Yes |
| 8 | CCND2 |  |  | 32 | 0.269 | 0.1051 | Yes |
| 9 | RPL10 |  |  | 33 | 0.265 | 0.1173 | Yes |
| 10 | RPL18 |  |  | 36 | 0.256 | 0.1288 | Yes |
| 11 | RPL41 |  |  | 42 | 0.249 | 0.1397 | Yes |
| 12 | HES6 |  |  | 44 | 0.248 | 0.1510 | Yes |
| 13 | DLL3 |  |  | 49 | 0.246 | 0.1618 | Yes |
| 14 | RPS8 |  |  | 51 | 0.245 | 0.1729 | Yes |
| 15 | RPL4 |  |  | 60 | 0.237 | 0.1828 | Yes |
| 16 | RPL28 |  |  | 62 | 0.235 | 0.1935 | Yes |
| 17 | BTG2 |  |  | 63 | 0.234 | 0.2043 | Yes |
| 18 | RPL35A |  |  | 67 | 0.231 | 0.2145 | Yes |
| 19 | RPS25 |  |  | 84 | 0.223 | 0.2229 | Yes |
| 20 | RPS27L |  |  | 91 | 0.218 | 0.2322 | Yes |
| 21 | RPS5 |  |  | 92 | 0.218 | 0.2422 | Yes |
| 22 | RPS16 |  |  | 103 | 0.212 | 0.2508 | Yes |
| 23 | HNRNPA1 |  |  | 108 | 0.209 | 0.2599 | Yes |
| 24 | TP53 |  |  | 116 | 0.205 | 0.2685 | Yes |
| 25 | RPL12 |  |  | 121 | 0.203 | 0.2774 | Yes |
| 26 | RPS27A |  |  | 136 | 0.198 | 0.2848 | Yes |
| 27 | RPS24 |  |  | 137 | 0.197 | 0.2939 | Yes |
| 28 | CCNB1IP1 |  |  | 142 | 0.196 | 0.3024 | Yes |
| 29 | MDM2 |  |  | 155 | 0.189 | 0.3097 | Yes |
| 30 | TMSB4X |  |  | 162 | 0.187 | 0.3176 | Yes |
| 31 | RPL36 |  |  | 166 | 0.187 | 0.3258 | Yes |
| 32 | RPS18 |  |  | 185 | 0.181 | 0.3320 | Yes |
| 33 | RPS15 |  |  | 193 | 0.178 | 0.3394 | Yes |
| 34 | BTF3 |  |  | 194 | 0.178 | 0.3475 | Yes |
| 35 | HIGD2A |  |  | 197 | 0.177 | 0.3554 | Yes |
| 36 | GLUL |  |  | 199 | 0.177 | 0.3634 | Yes |
| 37 | RPL8 |  |  | 215 | 0.170 | 0.3695 | Yes |
| 38 | RPSA |  |  | 219 | 0.169 | 0.3769 | Yes |
| 39 | RGS16 |  |  | 239 | 0.164 | 0.3822 | Yes |
| 40 | RPL13A |  |  | 255 | 0.161 | 0.3879 | Yes |
| 41 | GTF2I |  |  | 284 | 0.154 | 0.3917 | Yes |
| 42 | HNRNPC |  |  | 331 | 0.147 | 0.3930 | Yes |
| 43 | EIF3E |  |  | 334 | 0.146 | 0.3995 | Yes |
| 44 | CORO1C |  |  | 345 | 0.145 | 0.4050 | Yes |
| 45 | COMMD6 |  |  | 350 | 0.144 | 0.4111 | Yes |
| 46 | RPL23 |  |  | 457 | 0.127 | 0.4046 | Yes |
| 47 | SVIL |  |  | 471 | 0.126 | 0.4089 | Yes |
| 48 | TFDP2 |  |  | 507 | 0.122 | 0.4104 | Yes |
| 49 | H3F3B |  |  | 514 | 0.122 | 0.4153 | Yes |
| 50 | CHMP2A |  |  | 523 | 0.121 | 0.4199 | Yes |
| 51 | NONO |  |  | 549 | 0.118 | 0.4224 | Yes |
| 52 | RPL21 |  |  | 554 | 0.118 | 0.4274 | Yes |
| 53 | HDAC9 |  |  | 574 | 0.116 | 0.4305 | Yes |
| 54 | C12orf57 |  |  | 609 | 0.113 | 0.4317 | Yes |
| 55 | NEDD4L |  |  | 615 | 0.113 | 0.4363 | Yes |
| 56 | RPS6 |  |  | 647 | 0.110 | 0.4377 | Yes |
| 57 | MEX3A |  |  | 660 | 0.109 | 0.4413 | Yes |
| 58 | HMGN2 |  |  | 679 | 0.108 | 0.4442 | Yes |
| 59 | EEF1D |  |  | 682 | 0.107 | 0.4489 | Yes |
| 60 | RIOK3 |  |  | 687 | 0.107 | 0.4533 | Yes |
| 61 | SUMO2 |  |  | 710 | 0.105 | 0.4555 | Yes |
| 62 | ZC3H15 |  |  | 716 | 0.104 | 0.4598 | Yes |
| 63 | SEZ6L |  |  | 733 | 0.103 | 0.4626 | Yes |
| 64 | NFE2L2 |  |  | 748 | 0.102 | 0.4657 | Yes |
| 65 | DNAJC8 |  |  | 832 | 0.096 | 0.4604 | Yes |
| 66 | PAK3 |  |  | 845 | 0.095 | 0.4634 | Yes |
| 67 | TEAD2 |  |  | 857 | 0.094 | 0.4664 | Yes |
| 68 | SCRN1 |  |  | 1040 | 0.084 | 0.4490 | No |
| 69 | MARCKS |  |  | 1081 | 0.081 | 0.4481 | No |
| 70 | GLO1 |  |  | 1161 | 0.077 | 0.4424 | No |
| 71 | ZNF462 |  |  | 1164 | 0.077 | 0.4457 | No |
| 72 | KAT6A |  |  | 1167 | 0.077 | 0.4490 | No |
| 73 | RFTN2 |  |  | 1196 | 0.075 | 0.4492 | No |
| 74 | BAZ2B |  |  | 1208 | 0.075 | 0.4514 | No |
| 75 | C19orf70 |  |  | 1253 | 0.073 | 0.4496 | No |
| 76 | UBAP2L |  |  | 1280 | 0.072 | 0.4499 | No |
| 77 | EHBP1 |  |  | 1296 | 0.071 | 0.4514 | No |
| 78 | ZKSCAN1 |  |  | 1303 | 0.071 | 0.4540 | No |
| 79 | PHB |  |  | 1374 | 0.068 | 0.4490 | No |
| 80 | SKP1 |  |  | 1403 | 0.067 | 0.4488 | No |
| 81 | MYO10 |  |  | 1545 | 0.062 | 0.4352 | No |
| 82 | CDK2AP1 |  |  | 1550 | 0.062 | 0.4376 | No |
| 83 | TFAP2C |  |  | 1552 | 0.062 | 0.4403 | No |
| 84 | CDC37L1 |  |  | 1568 | 0.062 | 0.4414 | No |
| 85 | TMEM158 |  |  | 1598 | 0.061 | 0.4408 | No |
| 86 | ZNF146 |  |  | 1614 | 0.060 | 0.4418 | No |
| 87 | TCF3 |  |  | 1619 | 0.060 | 0.4441 | No |
| 88 | MAP6 |  |  | 1689 | 0.057 | 0.4386 | No |
| 89 | JAKMIP2 |  |  | 1755 | 0.055 | 0.4336 | No |
| 90 | CSNK1A1 |  |  | 1786 | 0.054 | 0.4325 | No |
| 91 | PEX19 |  |  | 1815 | 0.053 | 0.4317 | No |
| 92 | CLASRP |  |  | 1816 | 0.053 | 0.4341 | No |
| 93 | CHD7 |  |  | 1953 | 0.049 | 0.4205 | No |
| 94 | ENC1 |  |  | 1970 | 0.049 | 0.4209 | No |
| 95 | SETBP1 |  |  | 1990 | 0.048 | 0.4209 | No |
| 96 | INSM1 |  |  | 2051 | 0.046 | 0.4160 | No |
| 97 | NFIX |  |  | 2055 | 0.046 | 0.4178 | No |
| 98 | EZR |  |  | 2077 | 0.046 | 0.4174 | No |
| 99 | C1orf61 |  |  | 2155 | 0.043 | 0.4104 | No |
| 100 | NAP1L1 |  |  | 2171 | 0.043 | 0.4106 | No |
| 101 | PDCD5 |  |  | 2215 | 0.041 | 0.4075 | No |
| 102 | GSTP1 |  |  | 2453 | 0.035 | 0.3814 | No |
| 103 | BAZ1A |  |  | 2495 | 0.033 | 0.3781 | No |
| 104 | OXR1 |  |  | 2533 | 0.032 | 0.3753 | No |
| 105 | POLR2L |  |  | 2539 | 0.032 | 0.3762 | No |
| 106 | LHX2 |  |  | 2562 | 0.032 | 0.3751 | No |
| 107 | DAAM1 |  |  | 2604 | 0.031 | 0.3717 | No |
| 108 | TMEM258 |  |  | 2608 | 0.031 | 0.3728 | No |
| 109 | MAD2L2 |  |  | 2621 | 0.030 | 0.3728 | No |
| 110 | IRGQ |  |  | 2625 | 0.030 | 0.3738 | No |
| 111 | SIPA1L2 |  |  | 2628 | 0.030 | 0.3750 | No |
| 112 | CELSR2 |  |  | 2741 | 0.028 | 0.3632 | No |
| 113 | RBPJ |  |  | 3008 | 0.022 | 0.3332 | No |
| 114 | VEZF1 |  |  | 3022 | 0.022 | 0.3327 | No |
| 115 | FOXN2 |  |  | 3044 | 0.021 | 0.3312 | No |
| 116 | IVNS1ABP |  |  | 3158 | 0.019 | 0.3188 | No |
| 117 | PSME4 |  |  | 3211 | 0.017 | 0.3136 | No |
| 118 | LINC00667 |  |  | 3258 | 0.016 | 0.3090 | No |
| 119 | DUSP12 |  |  | 3291 | 0.016 | 0.3059 | No |
| 120 | NSMCE1 |  |  | 3336 | 0.015 | 0.3015 | No |
| 121 | MED10 |  |  | 3424 | 0.012 | 0.2919 | No |
| 122 | SALL1 |  |  | 3467 | 0.012 | 0.2875 | No |
| 123 | SCHIP1 |  |  | 3493 | 0.011 | 0.2851 | No |
| 124 | PSMD5 |  |  | 3745 | 0.006 | 0.2561 | No |
| 125 | FBL |  |  | 3777 | 0.005 | 0.2527 | No |
| 126 | BCCIP |  |  | 3795 | 0.005 | 0.2510 | No |
| 127 | STAM |  |  | 3805 | 0.005 | 0.2501 | No |
| 128 | MLC1 |  |  | 3855 | 0.004 | 0.2446 | No |
| 129 | LZTS1 |  |  | 3897 | 0.002 | 0.2399 | No |
| 130 | NCOA5 |  |  | 3907 | 0.002 | 0.2390 | No |
| 131 | SYNRG |  |  | 3910 | 0.002 | 0.2388 | No |
| 132 | RGL1 |  |  | 3970 | 0.001 | 0.2320 | No |
| 133 | FDFT1 |  |  | 3979 | 0.001 | 0.2311 | No |
| 134 | GPN1 |  |  | 4027 | 0.000 | 0.2256 | No |
| 135 | IFT74 |  |  | 4114 | -0.002 | 0.2157 | No |
| 136 | ZNF232 |  |  | 4143 | -0.002 | 0.2125 | No |
| 137 | MED12L |  |  | 4162 | -0.002 | 0.2105 | No |
| 138 | ARID1A |  |  | 4193 | -0.003 | 0.2071 | No |
| 139 | FGD4 |  |  | 4236 | -0.004 | 0.2024 | No |
| 140 | MDK |  |  | 4311 | -0.005 | 0.1940 | No |
| 141 | CCT5 |  |  | 4370 | -0.006 | 0.1875 | No |
| 142 | C8orf59 |  |  | 4471 | -0.008 | 0.1762 | No |
| 143 | LDHB |  |  | 4572 | -0.010 | 0.1651 | No |
| 144 | NOL4L |  |  | 4661 | -0.012 | 0.1553 | No |
| 145 | FLNB |  |  | 4682 | -0.012 | 0.1536 | No |
| 146 | RRP8 |  |  | 4808 | -0.014 | 0.1396 | No |
| 147 | SMAD5 |  |  | 4873 | -0.016 | 0.1329 | No |
| 148 | ACLY |  |  | 4883 | -0.016 | 0.1326 | No |
| 149 | ZFHX4 |  |  | 4941 | -0.017 | 0.1267 | No |
| 150 | LIMD1 |  |  | 4986 | -0.018 | 0.1224 | No |
| 151 | KIF26B |  |  | 4998 | -0.018 | 0.1220 | No |
| 152 | NKAIN3 |  |  | 5070 | -0.020 | 0.1146 | No |
| 153 | ADAR |  |  | 5098 | -0.020 | 0.1124 | No |
| 154 | CHMP1B |  |  | 5220 | -0.022 | 0.0993 | No |
| 155 | METAP2 |  |  | 5299 | -0.024 | 0.0913 | No |
| 156 | NPM1 |  |  | 5412 | -0.027 | 0.0795 | No |
| 157 | MEGF10 |  |  | 5415 | -0.027 | 0.0804 | No |
| 158 | ACTB |  |  | 5477 | -0.028 | 0.0746 | No |
| 159 | PRDX1 |  |  | 5508 | -0.029 | 0.0724 | No |
| 160 | MAGI1 |  |  | 5589 | -0.030 | 0.0645 | No |
| 161 | ENY2 |  |  | 5645 | -0.032 | 0.0595 | No |
| 162 | ZHX3 |  |  | 5667 | -0.032 | 0.0585 | No |
| 163 | PGRMC2 |  |  | 5718 | -0.033 | 0.0542 | No |
| 164 | GOLGA4 |  |  | 5812 | -0.035 | 0.0449 | No |
| 165 | ACIN1 |  |  | 5901 | -0.036 | 0.0363 | No |
| 166 | POU3F2 |  |  | 6000 | -0.038 | 0.0266 | No |
| 167 | GPRC5B |  |  | 6049 | -0.039 | 0.0228 | No |
| 168 | AGO1 |  |  | 6108 | -0.040 | 0.0179 | No |
| 169 | CACUL1 |  |  | 6184 | -0.042 | 0.0111 | No |
| 170 | USP3 |  |  | 6344 | -0.046 | -0.0054 | No |
| 171 | PTPRS |  |  | 6439 | -0.048 | -0.0141 | No |
| 172 | FANCF |  |  | 6465 | -0.049 | -0.0148 | No |
| 173 | CBFA2T2 |  |  | 6488 | -0.049 | -0.0151 | No |
| 174 | POU2F1 |  |  | 6515 | -0.050 | -0.0158 | No |
| 175 | EGR1 |  |  | 6519 | -0.050 | -0.0139 | No |
| 176 | MED27 |  |  | 6547 | -0.051 | -0.0147 | No |
| 177 | SDC2 |  |  | 6588 | -0.053 | -0.0169 | No |
| 178 | SRSF7 |  |  | 6593 | -0.053 | -0.0150 | No |
| 179 | DENND1B |  |  | 6613 | -0.053 | -0.0148 | No |
| 180 | TMTC2 |  |  | 6661 | -0.054 | -0.0177 | No |
| 181 | ETF1 |  |  | 6699 | -0.056 | -0.0195 | No |
| 182 | MMADHC |  |  | 6708 | -0.056 | -0.0179 | No |
| 183 | SCAND1 |  |  | 6709 | -0.056 | -0.0153 | No |
| 184 | FRMD4A |  |  | 6772 | -0.058 | -0.0198 | No |
| 185 | ARMCX6 |  |  | 6814 | -0.059 | -0.0219 | No |
| 186 | HEG1 |  |  | 6840 | -0.060 | -0.0221 | No |
| 187 | ACTR6 |  |  | 6860 | -0.061 | -0.0215 | No |
| 188 | EIF3A |  |  | 6999 | -0.065 | -0.0346 | No |
| 189 | TMEM98 |  |  | 7034 | -0.066 | -0.0356 | No |
| 190 | AASS |  |  | 7119 | -0.068 | -0.0422 | No |
| 191 | QSER1 |  |  | 7135 | -0.069 | -0.0408 | No |
| 192 | IST1 |  |  | 7142 | -0.069 | -0.0384 | No |
| 193 | UBE2O |  |  | 7231 | -0.072 | -0.0453 | No |
| 194 | SRRM2 |  |  | 7389 | -0.077 | -0.0601 | No |
| 195 | GDI2 |  |  | 7412 | -0.078 | -0.0591 | No |
| 196 | ZNF253 |  |  | 7494 | -0.081 | -0.0648 | No |
| 197 | SYNE2 |  |  | 7598 | -0.086 | -0.0729 | No |
| 198 | ZEB1 |  |  | 7621 | -0.087 | -0.0715 | No |
| 199 | DPF2 |  |  | 7623 | -0.087 | -0.0676 | No |
| 200 | DNM2 |  |  | 7685 | -0.090 | -0.0706 | No |
| 201 | PPME1 |  |  | 7729 | -0.093 | -0.0713 | No |
| 202 | PKM |  |  | 7732 | -0.093 | -0.0673 | No |
| 203 | LRP8 |  |  | 7752 | -0.093 | -0.0653 | No |
| 204 | PTTG1IP |  |  | 7790 | -0.095 | -0.0652 | No |
| 205 | PSMA3 |  |  | 7871 | -0.100 | -0.0700 | No |
| 206 | GPC2 |  |  | 7878 | -0.100 | -0.0661 | No |
| 207 | CA12 |  |  | 7910 | -0.102 | -0.0650 | No |
| 208 | CACNA2D1 |  |  | 7985 | -0.106 | -0.0688 | No |
| 209 | CELSR1 |  |  | 8013 | -0.108 | -0.0670 | No |
| 210 | ZNF292 |  |  | 8071 | -0.111 | -0.0685 | No |
| 211 | CTTN |  |  | 8073 | -0.112 | -0.0635 | No |
| 212 | FJX1 |  |  | 8110 | -0.115 | -0.0625 | No |
| 213 | GJC1 |  |  | 8132 | -0.116 | -0.0596 | No |
| 214 | PON2 |  |  | 8177 | -0.120 | -0.0592 | No |
| 215 | SLC4A7 |  |  | 8273 | -0.129 | -0.0644 | No |
| 216 | FABP5 |  |  | 8414 | -0.148 | -0.0740 | No |
| 217 | MSMO1 |  |  | 8505 | -0.162 | -0.0770 | No |
| 218 | GON4L |  |  | 8509 | -0.163 | -0.0699 | No |
| 219 | COL11A1 |  |  | 8518 | -0.165 | -0.0632 | No |
| 220 | PNN |  |  | 8524 | -0.167 | -0.0561 | No |
| 221 | SEC11A |  |  | 8542 | -0.171 | -0.0503 | No |
| 222 | PAXBP1 |  |  | 8573 | -0.179 | -0.0455 | No |
| 223 | RPN2 |  |  | 8716 | -0.256 | -0.0504 | No |
| 224 | LAPTM4A |  |  | 8730 | -0.267 | -0.0396 | No |
| 225 | TMX1 |  |  | 8738 | -0.275 | -0.0278 | No |
| 226 | CD63 |  |  | 8776 | -0.335 | -0.0168 | No |
| 227 | SLC1A3 |  |  | 8788 | -0.413 | 0.0009 | No |
Table: GSEA details [plain text format]

  

Fig 2: RICHARDS\_NOWAKOWSKI\_SCIENCE\_2017\_IPC\_NEN3\_UPREG: Random ES distribution      
 Gene set null distribution of ES for **RICHARDS\_NOWAKOWSKI\_SCIENCE\_2017\_IPC\_NEN3\_UPREG**
